# Advances and Challenges of Using the Sterile Insect Technique for the Management of Pest Lepidoptera

**DOI:** 10.3390/insects10110371

**Published:** 2019-10-25

**Authors:** František Marec, Marc J. B. Vreysen

**Affiliations:** 1 Biology Centre, Czech Academy of Sciences, Institute of Entomology, Branišovská 31, 37005 České Budĕjovice, Czech Republic; 2 Insect Pest Control Laboratory, Joint FAO/IAEA Division of Nuclear Techniques in Food and Agriculture, International Atomic Energy Agency, P.O. Box 100, 1400 Vienna, Austria; M.Vreysen@iaea.org

**Keywords:** SIT, inherited sterility, moths, cytogenetics, pest control programs, quality control, genetic sexing

## Abstract

Over the past 30 years, the sterile insect technique (SIT) has become a regular component of area-wide integrated pest management (AW-IPM) programs against several major agricultural pests and vectors of severe diseases. The SIT-based programs have been especially successful against dipteran pests. However, the SIT applicability for controlling lepidopteran pests has been challenging, mainly due to their high resistance to the ionizing radiation that is used to induce sterility. Nevertheless, the results of extensive research and currently operating SIT programs show that most problems with the implementation of SIT against pest Lepidoptera have been successfully resolved. Here, we summarize the cytogenetic peculiarities of Lepidoptera that should be considered in the development and application of SIT for a particular pest species. We also discuss the high resistance of Lepidoptera to ionizing radiation, and present the principle of derived technology based on inherited sterility (IS). Furthermore, we present successful SIT/IS applications against five major lepidopteran pests, and summarize the results of research on the quality control of reared and released insects, which is of great importance for their field performance. In the light of new research findings, we also discuss options for the development of genetic sexing strains, which is a challenge to further improve the applicability of SIT/IS against selected lepidopteran pests.

## 1. Introduction

The sterile insect technique (SIT) is an environment-friendly, autocidal method to manage insect pests on an area-wide basis [1]. To be applied against any pest, the SIT requires colonization and mass rearing of the target insect at reasonable cost, the sterilization of large numbers of the reared insects by ionizing irradiation using gamma- or X-rays, and their subsequent periodic release into the target area where they have to compete with wild males for matings with wild females. Virgin wild females that mate with released sterile males have no offspring, which in turn leads to suppression of the pest populations [2]. There are several examples of the successful long-term application of the SIT for the management of key dipteran pests, either of agricultural, veterinary, or medical importance [3,4,5,6]. However, many considered this pest control tactic less effective for moths (Lepidoptera) in view of their high resistance to ionizing radiation requiring high doses of gamma- or X-rays to induce complete sterility. These fully sterilizing radiation doses cause, besides desired genetic damage, various physiological defects that reduce the ability of sterile moths to compete with wild ones [7,8].

Interest in the use of radiation-induced sterility for the management of pest Lepidoptera was restored after finding that males of the codling moth *Cydia pomonella* (L.), irradiated with substerilizing doses, show a better competitiveness and transmit sterility to the next generation [9]. The so-called F_1_ sterility or inherited sterility (IS) was later found to be a characteristic feature of Lepidoptera [10,11]. This finding prompted numerous investigations in many lepidopteran pests, ranging from studies on radiation biology and the optimization of radiation doses through genetic principle of radioresistance, improving mass rearing and the competitiveness of irradiated moths, and increasing the efficacy of pest control to population modeling of the applicability of SIT versus IS and field release experiments [8,12,13]. These studies showed the great potential of SIT/IS for the population suppression and/or local eradication of a number of lepidopteran pests. To date, the SIT and IS techniques have been successfully implemented in control programs against several moth species in four countries from three different continents [13].

In this review, we point out the specific cytogenetic and cytological features of Lepidoptera, which should be taken into account in the research and application of SIT or IS. We discuss the high radioresistance and inherited sterility in Lepidoptera, provide an overview of the successful SIT/IS control programs against major lepidopteran pests, and present quality control measures for the released insects. We also address challenges resulting from new research findings.

## 2. Specific Cytogenetic and Cytological Features of Lepidoptera Relevant to SIT

Moths and butterflies (Lepidoptera) exhibit several peculiar cytogenetic and cytological characteristics that distinguish them from the other insects. These peculiarities are one of the main causes of the high resistance of Lepidoptera to ionizing radiation. They also play a crucial role in the mechanism of inherited sterility and their knowledge is essential for the development of genetic sexing strains. Understanding these peculiarities is therefore important for the successful implementation of SIT against a particular pest. The main differentiating features of Lepidoptera are (i) female heterogamety, which is associated with the achiasmatic mode of female meiosis, (ii) the holokinetic structure of chromosomes, which significantly contributes to the radioresistance, and (iii) dichotomous spermatogenesis, which is closely related to the competitiveness of males.

**Figure 1:**
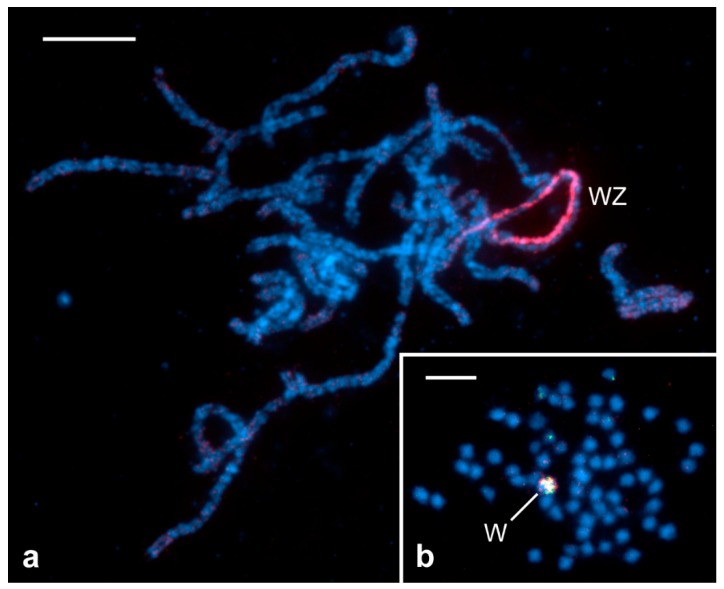
WZ sex chromosome system in Lepidoptera. Chromosomes were stained with DAPI (4’,6-diamidino-2-phenylindole; blue). (**a**) Pachytene oocyte of the codling moth, *Cydia pomonella* (L.), showing *n* = 28 meiotic chromosome bivalents; the neo-sex chromosome bivalent (WZ) is identified by fluorescence in situ hybridization (FISH) with W-painting probe (red), highlighting the W-chromosome thread [14]. (**b**) Mitotic oogonial metaphase of the cabbage moth, *Mamestra brassicae* (L.), showing 2*n* = 62 chromosomes [15]; the W chromosome is identified by comparative genomic hybridization (CGH) with both the female genomic DNA probe (green) and the male genomic DNA probe (red), resulting in yellowish labeling. Bar = 10 µm for (**a**) and 5 µm for (**b**).

Female heterogamety refers to a sex chromosome system of the WZ or Z0 type, where female gametes decide on the sex of the embryo. In insects, female heterogamety is a characteristic trait only for two sister orders, Trichoptera (caddisflies) and Lepidoptera. While caddisflies have a Z0/ZZ system (female/male), females of most lepidopteran species have a WZ sex chromosome pair [16]. The exceptions are species with multiple W and/or Z chromosomes and species without the W chromosome. The W absence is typical for basal lineages of Lepidoptera such as Micropterigidae, from which it is inferred that the Z0/ZZ system is an ancestral feature of both orders, Trichoptera and Lepidoptera, and the W chromosome is a later acquisition of Lepidoptera [17,18,19]. Moreover, sporadic losses of the W chromosome were found in a few species from different phylogenetic lineages of Lepidoptera where the W is present such as in wild silkmoths, *Samia cynthia* (Drury) [18,20]. However, the vast majority of economically important pests have a standard WZ/ZZ sex chromosome system [15,16,21,22] or, as reported recently for the family Tortricidae, neo-sex chromosomes, which arose by the fusion of the ancestral Z chromosome (and probably also the W chromosome) with an autosome (Figure 1a) [23,24,25]. This information is crucial for the development of genetic sexing strains for the SIT, as discussed further below.

**Figure 2:**
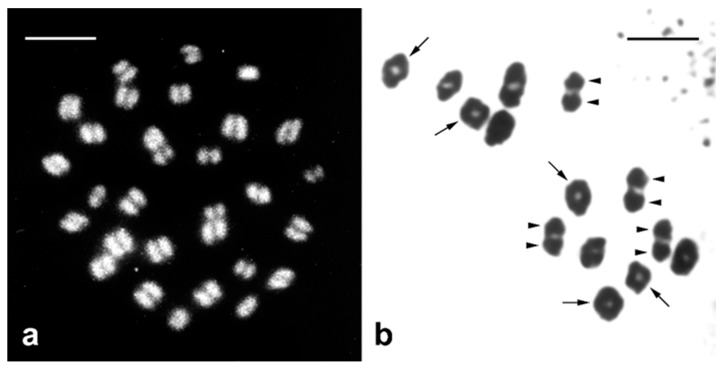
Comparison of female and male meiotic chromosomes in Lepidoptera. (**a**) Female metaphase I bivalents (*n* = 30) of the Mediterranean flour moth, *Ephestia kuehniella* Zeller, stained with DAPI (white). Each bivalent consists of two homologous chromosomes arranged parallel to each other; the proteinaceous structure of the modified synaptonemal complex (SC) forms an unstained gap between the two homologues. (**b**) Male metaphase I bivalents (*n* = 14) of the vaporer, *Orgyia antiqua* (L.), stained with lactic acetic orcein. Two homologous chromosomes are maintained in bivalents by chiasmata; two arrowheads indicate two homologues of bivalents with one chiasma, arrows indicate bivalents with two chiasmata. Bar = 5 µm.

Female heterogamety is associated with the achiasmatic mode of female meiosis. Lepidopteran oocytes undergo normal meiosis until the pachytene stage, when their chromosomes pair through the synaptonemal complex (SC) to form bivalents. However, then meiosis proceeds without recombination and chiasma formation, and the bivalents are maintained by modified SCs until chromosome segregation (Figure 2a) [26,27]. In contrast to many XY systems, the W and Z sex chromosomes pair completely during meiosis and form a regular bivalent, although they are largely non-homologous and often differ in size (Figure 1a) [14]. Males have a normal course of meiosis, including recombination followed by chiasma formation (Figure 2b) [28,29].

**Figure 3:**
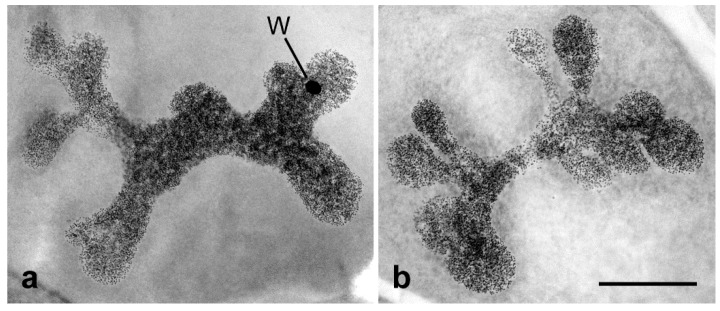
Highly polyploid nuclei of the Malpighian tubule cells from the carob moth, *Ectomyelois ceratoniae* Zeller, stained with lactic acetic orcein. (**a**) A nucleus from adult female showing a sex chromatin body (W). (**b**) A nucleus from male larva without sex chromatin. Bar = 50 µm.

The W chromosome, which is largely formed by heterochromatin, is responsible for another peculiarity of lepidopteran genomes. In the somatic interphase nuclei of females, it forms a deeply stained body, known as sex chromatin or W chromatin [30]. The sex chromatin is particularly conspicuous in the highly polyploid nuclei found in some tissues such as Malpighian tubules and silk glands, where it forms a large ball-shaped body (or bodies) composed of several hundreds to thousands copies of the W chromosome (Figure 3a,b) [31]. This female-specific trait is easily applicable as a marker to determine the sex of embryos and larvae [32] and also to identify sex chromosome aberrations in mutagenesis screens [30]. Sex chromatin bodies can also be isolated, for example using laser microdissection, to collect the W chromosome DNA either for the preparation of W-painting probes or for sequence analysis [14,33].

Chromosomes in Lepidoptera are usually small, numerous, and uniform in shape (Figure 1b). They lack a distinct primary constriction (the centromere) and their sister chromatids separate by parallel disjunction during mitotic metaphase [34]. During cell division, the spindle microtubules attach to a large kinetochore plate covering most of the chromosome surface [11,35]. These characteristics are in line with the criteria for holokinetic chromosomes [36]. The holokinetic nature of lepidopteran chromosomes is expected to facilitate karyotype evolution mainly via chromosomal fusion and fission by reducing the risk of formation of dicentric and acentric chromosomes [11]. However, recent results of comparative genomics along with previous cytogenetic data revealed surprising evolutionary stability of lepidopteran karyotypes, with most species having haploid chromosome numbers ranging from *n* = 28 to *n* = 32 and the most common and probably also ancestral number of *n* = 31 [37,38,39,40,41]. This remarkable stability contrasts with great diversity of karyotypes in some lepidopteran taxa, such as butterflies of the families Lycaenidae and Pieridae, due to chromosome fusion or fission [42,43]. More importantly, most pest species retain relatively conserved karyotypes with 2*n* = 28–31 [15,22,24,44,45,46].

**Figure 4:**
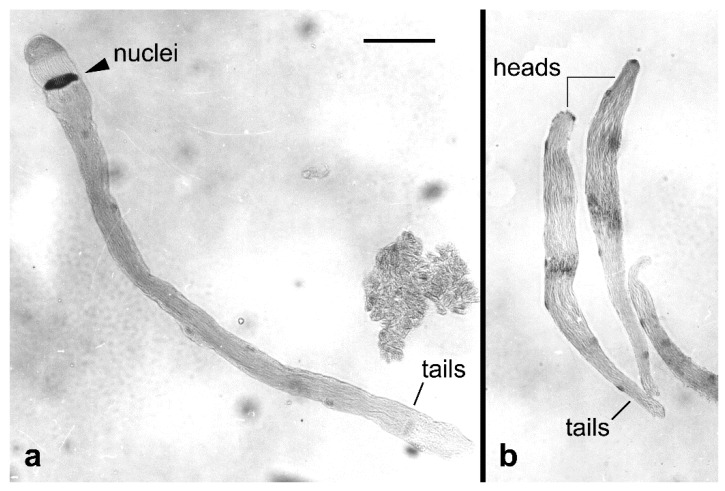
Sperm bundles in the Mediterranean flour moth, *Ephestia kuehniella* Zeller, stained with lactic acetic orcein. (**a**) A eupyrene sperm bundle. (**b**) Two apyrene sperm bundles. Bar = 100 µm.

Another peculiarity of Lepidoptera, the so-called dichotomous spermatogenesis was thoroughly reviewed in a comprehensive article earlier [47]. This trait is an evolutionary novelty as it occurs in all Lepidoptera except the basal genus *Micropterix*, but not in the sister order Trichoptera. Briefly, lepidopteran males produce two different types of sperm, the larger, nucleate and fertile eupyrene sperm (Figure 4a), and the smaller, anucleate and non-fertile apyrene sperm (Figure 4b). Both types are transferred to the female during copulation, however, eupyrene sperm remain in bundles and are less abundant (typically comprise 10–15% of the total sperm transferred), whereas apyrene sperm dissociate before male ejaculation [48]. The function of apyrene sperm has been the subject of many debates, but without a clear conclusion [47]. However, results of some studies suggest that the apyrene sperm maximize the males’ reproductive success by delaying female remating [49,50], which makes them important for the competitiveness of males, one of the most studied parameters when implementing SIT [13]. In addition, recent results in the silkworm, *Bombyx mori L.*, showed that apyrene sperm are essential for the migration of eupyrene sperm from the bursa copulatrix to the spermatheca and hence necessary for successful fertilization [51].

## 3. Resistance of Lepidoptera to Irradiation

**Figure 5:**
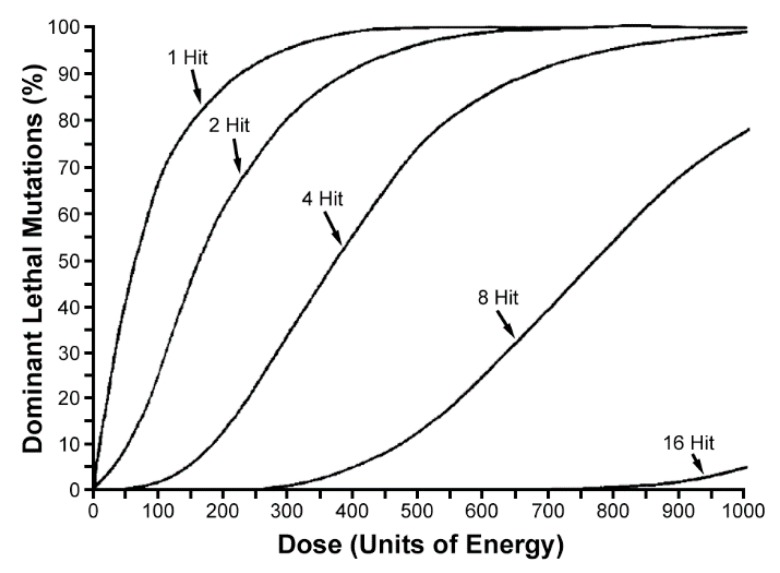
Computer-simulated curves for the induction of dominant lethal mutations in insect sperm. Shape of the curve changes as the number of hits required for a dominant lethal event increases. (Reproduced from [55] with permission of Elsevier).

In the SIT, sterility is primarily the result of dominant lethal mutations (DLMs) caused by ionizing radiation in germ cells. DLMs mostly arise as a consequence of chromosomal aberrations that after fertilization result in the formation of anaphase chromosome bridges indicating the presence of dicentric chromosomes, the occurrence and subsequent loss of chromosome fragments, and other abnormalities in the dividing nuclei, resulting in the death of the zygote or the embryo [7]. In most insects, DLMs are expressed during early embryogenesis, but in Lepidoptera, no chromosomal bridges are evident in the cleavage nuclei, and the majority of DLMs are expressed very late in embryonic development [52,53]. In addition, chromosome fragments may persist for a number of mitotic cell divisions, and can even be transmitted through germ cells to the next generation [54]. Dose–response curves, developed for the induction of DLMs in the mature sperm of lepidopteran males, approximated S-shape curves for 8–16-hit kinetics, while in Diptera, the curve displayed a 1-hit curve (Figure 5) [55,56]. The results support a hypothesis that in lepidopteran males, in contrast to dipteran flies, multiple chromosome rearrangements must be induced to be expressed as DLMs. These data illustrate why such high radiation doses (350–500 Gy) are required for lepidopteran males to be completely sterile [55]. For comparison, fully sterilizing doses for tephritid fruit fly males were determined to range from 44 to 83 Gy with a mean of 63 Gy (based on 21 species) [57,58]. However, the germ cells of female Lepidoptera are much more sensitive to irradiation than the sperm of males. The apparent reason is the developmental stage of germ cells. In lepidopteran insects, which are usually irradiated as mature pupae or freshly emerged adults, female meiosis is arrested at metaphase I in the nuclei of mature oocytes, and does not proceed until the eggs have been oviposited, while males already have mature sperm [28]. In females, irradiation may thus disrupt the normal course of meiosis including chromosome segregation and in addition, cause various secondary damage due to the large amount of cytoplasm in mature oocytes. Therefore, doses of 100–200 Gy are sufficient to achieve almost full sterility in irradiated females, as found in a number of lepidopteran pests examined [11,13].

The high resistance of Lepidoptera to ionizing radiation is a complex trait, reflecting the joint effects of several intracellular mechanisms and genome characteristics. As previously found, the cultured lepidopteran cells are 50–100 times more resistant to radiation-induced death than cultured mammalian cells, whereas dipteran cells are only 3–9 times more resistant [59,60]. Based on the results of experiments conducted to understand the molecular mechanisms of the radioresistance of the cultured cells, it has been proposed that Lepidoptera may have an inducible cell recovery system and more efficient DNA repair [59]. Moreover, recent studies in the Sf9 cell line, originally derived from the fall armyworm *Spodoptera frugiperda* (J.E. Smith), revealed an unusually low level of radiation-induced apoptosis, supported by an efficient antioxidant defense system and a high activity of histone deacetylases, both contributing to the radioresistance of lepidopteran cells [61,62].

**Figure 6:**
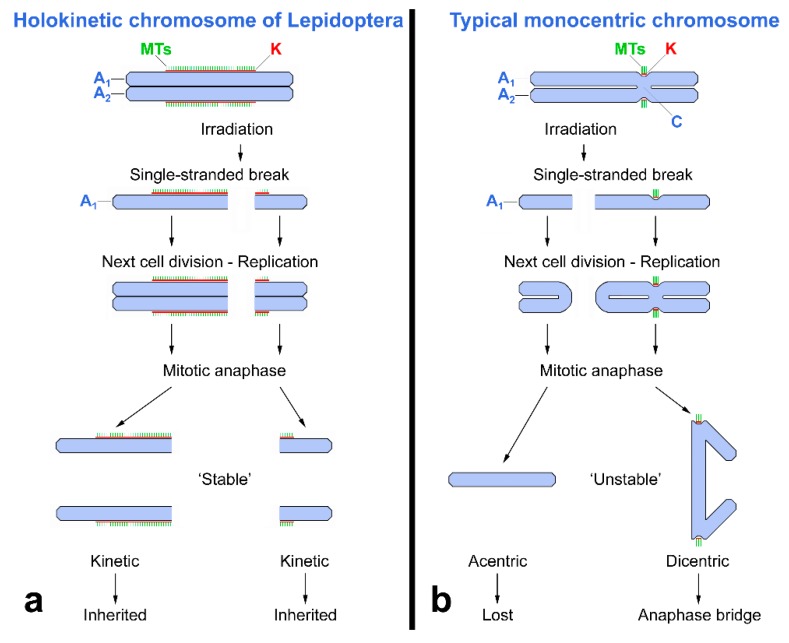
Comparison of structure of holokinetic and monocentric mitotic chromosomes and consequences of chromosome breakage. (**a**) Holokinetic chromosome of Lepidoptera with two sister chromatids (A_1_ and A_2_), each with a large kinetochore plate (K) covering about 50% of the chromosome surface; spindle microtubules (MTs) are attached to the kinetochore. (**b**) Typical monocentric chromosome, where sister chromatids are linked in a primary constriction, the centromere (C); the kinetochore (K) is localized on the surface of the centromere (modified according to [11]).

A significant role in the radioresistance of Lepidoptera can be attributed to the holokinetic structure of their chromosomes [7,8]. As noted above, lepidopteran chromosomes possess a large kinetochore plate that covers a significant portion of the poleward chromosome surface [35]. The large binding site for spindle microtubules during cell division ensures that most radiation-induced breaks do not lead to the loss of chromosome fragments, in contrast to species with typical monocentric chromosomes [11]. The large kinetochore plates also reduce the risk of the formation of dicentric chromosomes, acentric fragments, and other unstable aberrations that would lead to DLMs (Figure 6a,b) [63]. Moreover, chromosome fragments, if they have retained a part of the kinetochore plate to be attached to the spindle, can be inherited for many generations, as demonstrated in the Mediterranean flour moth, *Ephestia kuehniella* Zeller [54].

## 4. Inherited Sterility in Lepidoptera

The term ‘inherited sterility’ (IS) or ‘F_1_ sterility’ has been used for a phenomenon that occurs when lepidopteran species are treated with substerilizing doses of ionizing radiation so that they are only partly sterile, but their F_1_ offspring exhibit a higher level of sterility than the irradiated parents. In addition, radiation-induced deleterious effects can be inherited for several generations [11]. Since females are more radiosensitive than males [64,65], it is possible to select a radiation dose at which females are almost completely sterile, while the males are only partially sterile. This makes IS very appropriate for pest control programs, because lower doses of radiation increase the quality and competitiveness of the released insects [13]. Another advantage of IS compared to SIT is its much better compatibility with other pest control strategies, such as the use of insect pathogens, synthetic pheromones, and parasitoids [11].

IS was first reported in the silkworm, *Bombyx mori* L. [66], and then confirmed in the wax moth, *Galleria mellonella* (L.) [67], but the results of these early studies on radiation biology in lepidopteran models were forgotten. Much later, the phenomenon of IS was rediscovered in the codling moth, *C. pomonella*, by Proverbs [9], who also proposed using it for codling moth control instead of SIT. His results, together with Knipling’s theoretical model [68], prompted numerous investigations in many lepidopteran pests. A historical overview on IS research along with a list of studied species has been provided in a comprehensive review [11]. The genetic basis of IS has also been reviewed and discussed earlier [8,10,11]. No significant progress has been made since then in understanding the principle of IS. Yet, for a complete overview, we summarize our current knowledge below.

Several previous studies demonstrated a high incidence of chromosomal aberrations in the F_1_ progeny of irradiated males using light microscopy [11]. However, these studies examined only male metaphase I chromosomes, in which light microscopy lacks resolution to identify types of aberrations. A recent study, examining mitotic metaphases and pachytene spermatocytes alongside metaphase I in F_1_ males of the tomato leafminer, *Tuta absoluta* (Meyrick), reported that the irradiation of male parents with a substerilizing dose of 200 Gy induced mainly chromosomal translocations and fragmentation of the chromosomes [69]. However, the most valuable results were obtained using a modified microspreading technique for electron microscopy to study radiation-induced chromosome aberrations in F_1_ females and males of the flour moth [63]. This technique enabled the high resolution and classification of chromosome aberrations in long pachytene chromosomes based on homologous pairing. In the F_1_ progeny of males irradiated with 100 Gy, 150 Gy, or 200 Gy, mainly various translocations (non-reciprocal, reciprocal, and multiple) and numerous chromosome fragments were identified, whereas interstitial deletions and inversions were rare. Based on the results of the dose–response analysis, it was concluded that translocations are most responsible for the production of unbalanced gametes in F_1_ progeny, and thus represent the main chromosomal mechanism of IS [63]. The study also showed that the F_1_ sterility predicted from observed aberration rates was much higher than the actual sterility reported earlier [56]. This finding suggests a mechanism favoring the balanced segregation of chromosomes. Such a mechanism could include the formation of modified SCs in the achiasmatic meiosis of females and the ability to invert the order of the main meiotic events in males. Both the modified SCs and the so-called inverted meiosis facilitate proper chromosome segregation and hence rescue the fertility of heterozygotes for chromosomal aberrations, as recently demonstrated in wood white butterflies of the genus *Leptidea* [70]. The above detailed analysis of radiation-induced chromosomal aberrations by electron microscopy [63] also offered an explanation of the higher IS found in F_1_ males than in F_1_ females, as reported in previous studies [11]. Three factors might contribute to this phenomenon: (i) the ability of males to survive more extensive damage to chromosomes than females, (ii) the occurrence of crossing-over during spermatogenesis, which might increase the number of unbalanced gametes produced by F_1_ males but not in the achiasmatic meiosis of females, and (iii) a higher impact of radiation-induced deleterious effects on the fertility of F_1_ males, such as defects in spermiogenesis or sperm transfer [69,71].

## 5. Successful Application of the SIT/IS Against Lepidopteran Pests

Both the SIT and the related IS technique [8,9,10] offer great potential as additional control tactics for integration with other control methods in area-wide integrated pest management (AW-IPM) approaches [1] against lepidopteran pests. In the last two decades, several successful operational programs have been testimony to the potential that this control tactic offers as an efficient addition to the existing control tool box. As with other insects, the SIT/IS can be applied against Lepidoptera using different strategic approaches, e.g., suppression, local eradication, and containment strategies [72], and the below provides a summary of some of the success stories in the last two decades.

### 5.1. False Codling Moth Suppressed in Eastern and Western Cape, South Africa

The false codling moth (FCM), *Thaumatotibia leucotreta* (Meyrick) (Tortricidae), is an indigenous moth of sub-Saharan Africa, where it infests a large number of wild and commercial plants. In South Africa, it is a phytosanitary pest, and has an extensive host range (88 plant species) with grapes, pomegranates, and citrus as the main targets. The moth has 5–6 generations/year in South Africa, and the females can oviposit up to 300 eggs, resulting in high-density populations until late in the citrus season. South Africa is the second biggest exporter of citrus in the world with about 1400 growers who, in 2013, exported around 106 million cartons of citrus. The FCM has been controlled traditionally using broad-spectrum insecticides, but biological control methods and mating disruption are also available. FCM has developed resistance to many of the commonly used insecticides, and as a result, the SIT has been developed for this pest as a viable additional control tactic [73,74].

**Figure 7:**
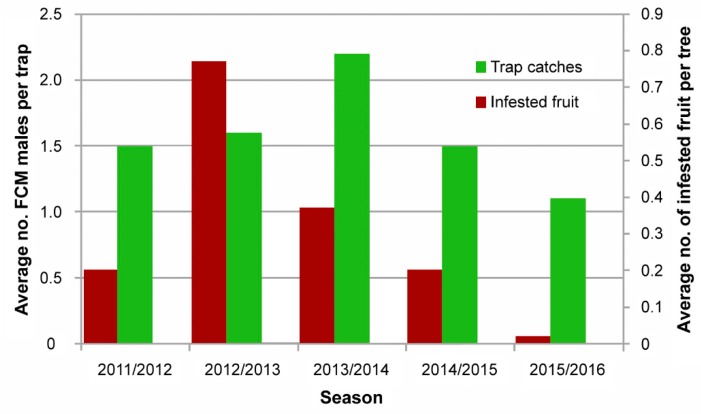
Wild false codling moth (FCM) trap catches and the average percentage of infested fruits per tree in the Sunday River Valley, Eastern Cape, South Africa, from 2011 to 2016 (reproduced with permission from Nevill Boersma, XSIT, Citrusdal, South Africa).

In 2005–2006, a pilot trial was implemented on a 35-ha orchard of Washington navel oranges in Citrusdal. One thousand 150 Gy-irradiated male and female moths were released from all-terrain vehicles twice a week for 28 weeks during the growing season. Sterile to wild male over-flooding ratios of 41:1 were obtained during the experiment, and at the end of the trial, crop losses due to the FCM infestation was reduced by 95.2% in the SIT-treated area as compared with the control orchard. As a result, the SIT for FCM was commercialized in South Africa, and a private company (XSIT) was established that in 2007 started releases of sterile moths over an area of 1500 ha in the Western Cape. This area has continuously expanded, reaching in 2016 8600 hectares in the Western Cape, 8900 hectares in the Eastern Cape, and 1200 ha in the Northern Cape. The success of this expansion is evidenced e.g., in the Eastern Cape by a reduction in the percentage of infested fruit per tree from 0.2–0.77 in the 2011/2012 and 2012/2013 season to 0.02 in the 2015/2016 season (Figure 7).

Several factors contributed to the success of the program, such as: (i) the development of resistance to insecticides in FCM that threatened the existence of the export-based industry, (ii) the citrus industry being a single-crop industry operated by a single organization (unlike the deciduous fruit industry), which facilitated obtaining and coordination of the necessary funds, and (iii) the industry had its own research entomologists, some of whom could devote their entire time to developing the SIT technology [75].

### 5.2. Codling Moth Suppressed in the Okanagan Valley of British Columbia, Canada

The codling moth, *Cydia pomonella* (L.) (Tortricidae), was a severe pest of apples and pears in the Okanagan Valley of BC, Canada where it was first recorded in 1900 [76]. Research in the 1960s and 1970s by Proverbs and colleagues [77,78] resulted in the development of the SIT for this pest, and ultimately culminated in a pilot trial (1976–1978) that showed the feasibility of integrating the use of insecticides with the release of sterile moths to suppress codling moth populations [79]. This paved the way to implement an operational program that would cover the entire valley. A mass-rearing facility was built in 1993 in Osoyoos (south of the valley) with a production capacity of 15 million sterile moths per week. The Okanagan Kootenay Sterile Insect Release (OKSIR) program was initiated in 1994 when the first sterile moths were released in the growing season. Although the program had the initial objective to eradicate codling moths from the valley, its scope changed to a suppression program in 1998 [76].

**Figure 8:**
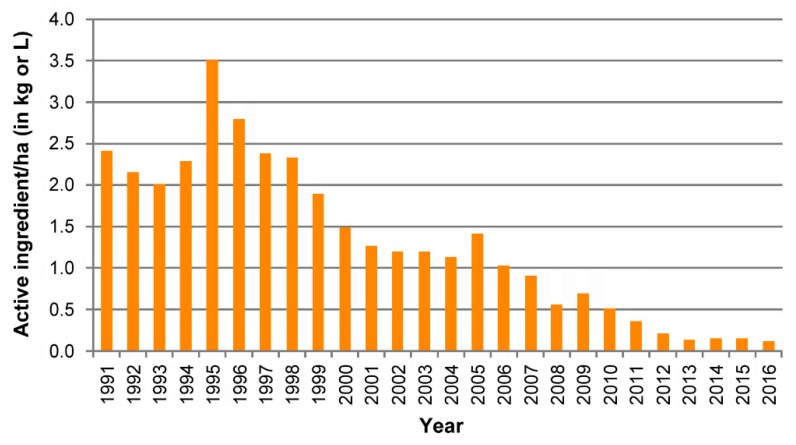
Estimates of pesticide use against the codling moth from 1991 to 2015 in the OKSIR Program area, Okanagan Valley. Sales data for the 15 products registered for the use against codling moth are used to determine these values. However, a number of these insecticides are also used against other pests and/or crops. The proportion of total sales of a given product used against the codling moth is estimated by local tree fruit experts (Jerry Vakenti, BC Ministry of Water, Land and Air Protection; Hank Markgraff, Head of Field Service, BC Tree Fruits; Hugh Philip, IPM specialist, Independent Consultant). The estimates of active ingredients are divided by the area (ha) of planted pome fruit in the program area to account for changes in sales due to the amount of pome fruit under cultivation. From 1991 to 2015, there was an estimated 96% reduction in pesticides used against the codling moth. Other factors, such as changes in application rates in spindle vs. traditional plantings, new product formulations, etc. contribute in part to this reduction (reproduced with permission from OKSIR).

The OKSIR program is the longest-running, most successful, area-wide integrated pest program for the suppression of codling moth in the world, and its implementation is accompanied by continuing extensive research [80,81,82,83,84,85]. The SIT is integrated with orchard sanitation, surveillance, tree banding, and mating disruption. After more than 20 years of operation, the codling moth populations in the valley have been drastically reduced, and as a result, the growers, the industry, and the local community have significantly reduced fruit damage and costs associated with codling moth control. The program has achieved less than 0.2% damage in more than 90% of all commercial pome fruit acreage and reduced insecticide use to control codling moth by over 95% in the valley (from 50,000 kg of chemicals in 1991 to <3000 kg in 2015; Figure 8). In addition, the number of chemical sprays targeting codling moth has been reduced from 1.5–2.7 sprays/acre in the early 1990s to <0.3 sprays/acre in 2013 in the southern part of the valley [86]. A recent cost–benefit analysis showed the economic efficiency of the program, i.e., a benefit to the producers from insecticide cost savings, monitoring cost savings and reduction in codling moth injury amounting to CAN $395/acre (versus CAN $377/acre for mating disruption). The economic benefits per acre of orchard were much higher using the OKSIR strategy as compared to using conventional insecticides: the overall cost–benefit ratio of the SIT program was 1.19 for the producer and 2.51 in total [87].

The food industry in the Okanagan believes that area-wide pest management should remain a priority [85], and OKSIR has been responding in this respect, and is assessing how the area-wide structure can be utilized to support the prevention and suppression of other newly invasive species, e.g., the apple clear wing moth *Synanthedon myopaeformis* (Borkhausen), the apple maggot *Rhagoletis pomonella* (Walsh), and the brown marmorated stink bug *Halyomorpha halys* Stål [88].

### 5.3. Pink Bollworm Eradication from Southern USA and Northern Mexico

The pink bollworm, *Pectinophora gossypiella* (Saunders) (Gelechiidae), is native to Asia, but has become an invasive pest in most of the world’s cotton-growing regions. It reached the cotton belt in the southern US by the 1920s, where it became one of its major pests. The female moth oviposits eggs in a cotton boll, and hatching larvae inflict damage by chewing through the cotton lint to feed on the seeds. As cotton is used for both fiber and seed oil, the damage is twofold. In addition, the rupture of the protective tissue around the boll is also a portal of entry for other insects and fungi.

The use of sterile moths against pink bollworm started as a containment program in 1968 to protect the cotton fields in the San Joaquin Valley of California. For more than 20 years, sterile moths were released every season, covering 0.4 million hectares of cotton that prevented the establishment of the pest [89].

However, economic losses caused by the pink bollworm to the industry in the US remained very high, i.e., to the order of USD 32 million/year in control costs and yield losses. As a result, in 2002, the industry initiated phase I of a larger eradication program that had the goal of eliminating this pest burden from the all cotton-producing areas of the US and adjacent areas of northern Mexico. The program was driven by the growers and the transition from one phase to the next was subject to a growers’ referendum. The program adopted an AW-IPM approach integrating extensive surveys, transgenic *Bt* cotton, mating disruption, and sterile moth releases. These control tactics were applied area-wide for a period of 4–5 years, i.e., *Bt* cotton was combined with trapping and mating disruption during the first year, and in some cases, during the second year. Thereafter, the program used *Bt* cotton, pheromones, and the daily releases of sterile insects to complete eradication [90].

The grower’s associations covered the cost of program operations, trapping, and pheromone applications, whereas the planting of *Bt* cotton was encouraged but left at the discretion of the growers who had to cover these expenses. The cost of sterile insect rearing and daily releases was provided by the US Department of Agriculture’s (USDA) Animal and Plant Health Inspection Service (APHIS) as cost-share to the program, and the mass-rearing facility was paid for by the California Cotton Producers and belonged to the California Department of Food and Agriculture. In general, 80% of the total program costs was covered by the growers and 20% was covered by the federal government. On 19 October 2018, the US Secretary of Agriculture officially declared the pink bollworm eradicated from the United States [91,92].

### 5.4. Cactus Moth Eradicated from Two Islands in Mexico

The cactus moth, *Cactoblastis cactorum* Berg (Pyralidae), is a pest of high economic and ecological importance for Mexico and the Southern US. Whereas Mexico remains now free of this pest, there is a high risk of invasion from some Caribbean Islands or from the US, where the pest was introduced in 1957 (voluntarily) and 1989 (accidentally in the Florida Keys), respectively [93]. The pest thrives on the cacti of the *Opuntia* genus, and it was used to great success as a biological control agent, e.g., in Australia, where approximately 25 million hectares of invasive *Opuntia* were eliminated by the cactus moth [94]. The spread of this moth along the south coast of the US raised major concerns such as potential harm to rare wild *Opuntia* species in a fragile desert ecosystem in the southwestern US and Mexico, where more than 3 million hectares are covered with wild *Opuntia* species [95,96]. In Mexico, *Opuntia* is an important economic commodity worth over USD 100 million per year, i.e., with 150,000 hectares of cacti cultivated for fodder, 60,000 hectares cultivated for the production of prickly pear, and 10,500 hectares cultivated for *Opuntia* leaf [95].

In July 2006 and May 2007, outbreaks of the pest were detected on the Isla Mujeres and the Isla Contoy, respectively, both located in southeastern Mexico. The Government of Mexico reacted swiftly, and a program to eradicate the cactus moth was put in place. The success of the program was related to several aspects such as the early detection of the pest before it could get established, and the adoption of an integrated approach that used the mechanical removal of egg sticks, host removal, and the release of sterile males (as no insecticides were allowed on the islands, which are protected areas). In accordance with nature protection requirements, the removal of host plants was not applied to endangered species of *Opuntia* and other cacti, except for the removal of plant parts (e.g., cladodes) that were infested with cactus moth larvae [97]. In March and October 2009, the government declared the pest eradicated from the Isla Mujeres and from the Isla Contoy (State of Quintana Roo), respectively [98].

### 5.5. Australian Painted Apple Moth Eradicated from New Zealand

From 1999 to 2004, the Australian painted apple moth *Teia anartoides* Walker (Lymantriidae) was the target of an eradication program in Auckland, New Zealand [99,100]. Female painted apple moths are flightless, and ballooning larvae are the main means of dispersal in this species [101]. The insect was considered to have potential for significant economic and ecological damage in New Zealand, because its host range includes plants of importance to horticulture and forestry, as well as to natural ecosystems. Estimates of its potential economic damage ranged from USD 30 to 213 million over 20 years [102].

Therefore, a mitigation program was initiated to eradicate the pest, and the Ministry of Agriculture and the Forestry Biosecurity Authority were the responsible authorities for its implementation. Starting in 2001, a monitoring program using traps with caged female moths was implemented to map the distribution of the pest. Although the pheromone had been identified [103], it proved to be highly unstable, and virgin females were used to bait traps throughout the program. All data were transferred into a geographic information system (GIS) to keep track of the spatial and temporal distribution and densities of the population, and to manage the aerial release of sterile insects [104]. The female moths were mass-reared for this purpose, and trap arrays of up to 2000 geo-referenced traps were serviced weekly. The geographic area of the infestation was relatively small, and confined to some 12,000 hectares [100].

By 2002, a male catch in a trap was followed immediately by the deployment of a higher density network of traps in order to locate the breeding population. A targeted program of ground searches was also used to define the area occupied by the pest, and host removal was used where possible [104].

The eradication program used an integrated approach combining insecticides (chlorpyrifos, deltamethrin) sprayed on host trees from the ground, the aerial application of the insecticidal pathogen *Bacillus thuringiensis* (Berliner), subsp. *kurstaki* (Btk) [105,106], and the SIT as part of an end game strategy, once the other tactics had lowered population densities [107]. Released moths were dispersed up to five kilometers, and 17% of irradiated males released were recaptured [100]. This indicated that irradiated insects were of adequate quality to compete with the native pest population.

From January 2003 until April 2004, the program released a total of 350,000 male moths that were irradiated as pupae with 100 Gy [100]. This rather low dose was chosen to increase the field competitiveness of the released insects. The releases were terminated 15 months after the last wild moth had been trapped.

## 6. Quality Control for Lepidoptera SIT or IS

It is well known that the SIT/IS can only be applied successfully when the released sterile insects can effectively compete with their wild counterparts for mating with wild females. Despite the many operational successes in the last years, expansion of the SIT/IS for lepidopteran pests could be facilitated through improved mass rearing and quality control, and the development of better protocols for handling, irradiation, shipping, release, and field assessments. In the last years, the Joint FAO/IAEA Division of Nuclear Techniques in Food and Agriculture has been fostering this type of research through two Coordinated Research Projects (CRP), i.e., “Increasing the Efficiency of Lepidoptera SIT by Enhanced Quality Control” (implemented between 2008 and 2014) [13], and a follow-up CRP entitled “Improved Field Performance of Sterile Male Lepidoptera to Ensure Success in SIT Programmes”, which has been implemented since 2016. The focus of the first CRP was on the identification of factors and variables that affect the quality and field performance of released moths, and validation of new tools and methods to assess and predict the field performance of sterile moths.

### 6.1. Research on Factors and Variables that Affect the Quality of Lepidoptera Used in SIT/IS Programs

The implementation of a number of operational AW-IPM programs against various insect pests has created more and more awareness that the mass rearing of insects for longer periods in an artificial environment imposes various selection pressures on the insects that can result in undesirable traits in field-released male insects. As a result, the reared insects are (often unwillingly or unnoticed) well adapted to the artificial environment, but not necessarily to the ecological requirements of the target zone, or do not have the best possible mating competitiveness. In addition, handling, irradiation, storage, transport, and release procedures may reduce the longevity and competitiveness of the released insects. In the past, mass-rearing programs have often emphasized quantity (numbers of insects produced) over quality (i.e., sexual competitiveness), and this philosophy sometimes resulted in lower program efficiency, e.g., requiring more insects to obtain the same level of suppression/progress or leading to an extended duration of the program, which entailed increased program costs [108].

Recently, significant progress has been made to better understand the factors and variables that affect the quality and the field performance of released moths. Several biological attributes (e.g., development rate, diapause, temperature adaptation, circadian rhythm, mating habits, flight capabilities) and operational components (cold storage, packaging and transport, release technology, and irradiation) were studied [13], and the below review describes some of the highlights.

The tolerance of released insects to thermal extremes may affect their activity and survival, and evidence has surfaced that host plants may mediate the lower critical thermal limits of some Lepidoptera [109]. Some laboratory-reared Lepidoptera, such as the African sugarcane borer *Eldana saccharina* Walker (Pyralidae) were more heat-tolerant than wild moths, and wild moths were more cold-tolerant than their laboratory-reared counterparts. Irradiation affected thermal tolerance, and moths irradiated with a lower dose were more heat and cold-tolerant than those irradiated with a higher dose [110]. So far, there has been no evidence found for cold hardening, and false codling moth survival could not be increased through exposure to non-lethal, low- and high-temperature pre-treatments [111]. However, significantly more low temperature-acclimated codling moths were recaptured under cooler conditions in the wild than either warm-acclimated or control moths [112].

The effect of irradiation on the quality of male moths was the topic of much research. In general, the hatching of eggs produced by untreated females that had mated with irradiated males decreased with increasing doses given to male moths. For IS programs, however, it is important to know the female sterilizing dose, as no genetic sexing systems exist to separate the female from the male moths, and the release of partially fertile females needs to be avoided. The radiation sensitivity of female moths appeared species-dependent, i.e., female codling moths were completely sterile when treated with a dose of 100 Gy [113], whereas a dose of 150 Gy was sufficient to completely sterilize females of *E. saccharina* and the European grape vine moth *Lobesia botrana* (Denis and Schiffermüller) (Tortricidae) [114,115]. Females of the litchi stem-end borer *Conopomorpha sinensis* Bradley (Gracillariidae) and the tomato leaf miner *T. absoluta* (Gelechiidae) were more radioresistant, and required 200 Gy for complete sterility [116,117,118], whereas eggs produced by females of the light brown apple moth (LBAM) *Epiphyas postvittana* (Walker) (Lymantriidae), which had been irradiated with 250 Gy, still showed 0.1% hatch [119]. A dose of 300 Gy was required to fully sterilize females of the date moth, *Ectomyelois ceratoniae* (Zeller) (Pyralidae) [120]. Rearing codling moths through diapause improved their competitiveness in orchards, but their radiosensitivity was similar to that of moths reared under non-diapause conditions [121]. Manipulations of atmospheric oxygen content may lower post-irradiation somatic damage while preserving sterility and improving sterile insect performance. Anoxia treatment of the cactus moth increased their flight performance, mating success, and longevity, while maintaining F_1_ male sterility at acceptable levels [122]. Anoxia treatment of irradiated cactus moth males lowered their mortality and extended their lifespan at each dose [123].

Whereas released sterile males need to be of a good biological quality to adequately compete with their wild counterpart [5,124], it is equally important that there are no mating barriers between the strain used for release and the wild population in the target area [125,126]. Mating studies in walk-in field cages showed the complete absence of mating barriers between codling moth populations from diverse regions of the world, i.e., Argentina, Armenia, Canada, Chile, New Zealand, Syria, and Switzerland [127]. In addition, 200-Gy irradiated or laboratory-reared *E. saccharina* showed no negative effects due to laboratory rearing or radiation treatment when tested against wild insects in field cages [128]. A substerilizing dose of 250 Gy administered to *C. sinensis* males would be adequate for programs that include an IS component based on competitiveness values obtained in field cages [118]. Releasing substerile *T. absoluta* males in field cages at a 15:1 substerile (200 Gy-treated pupae) to untreated male ratio caused a significant decline in larval production as compared with that in untreated control cages [117]. The radiation dose and mating frequency of LBAM was significantly negatively correlated [129], and the production of the sex pheromone by the females declined significantly with increasing doses of radiation. As a result, male catch in traps baited with 300 Gy-irradiated females was reduced to 11% of that in traps baited with non-irradiated control females.

Released sterile insects need to be sufficiently mobile, and knowledge about the mobility and dispersal characteristics of released insects is essential for developing and designing appropriate release strategies [124]. Experiments in a wind tunnel showed that the mobility of irradiated LBAM males toward a pheromone lure was significantly reduced in comparison with non-irradiated males [129]. Mark–release–recapture experiments with 250 Gy-irradiated male LBAM showed a reduced male moth recapture in hedgerows and in vineyards to >75% of control values [130]. Similarly, a flight assessment cage proved to be a valuable tool for measuring the quality of *L. botrana* moths, and a radiation dose of 350 Gy (but not 150 Gy) significantly lowered the flight responses of males to calling females in the cage [131]. In addition, in a field release, the performance of 150 Gy-irradiated males and untreated males were similar, whereas the dispersal of 350 Gy-irradiated males was reduced [131]. A flight mill is another useful tool to measure the mobility of moths. It was used to demonstrate that the flight distance, flight duration, and speed of 150–200 Gy-irradiated and untreated males of the litchi stem-end borer were not significantly different [132], which is a result that was confirmed in field release–recapture experiments.

### 6.2. Research on Tools and Methods to Assess Sterile Moth Field Performance

Available tools and methods to assess the quality of sterile males and link it to their field performance are scarce. Our understanding of how to assess and improve insect quality from the factory to the field requires an integration of laboratory, walk-in field cage, and open-field methods. The relation between the poor performance of insects in the field and how these insects perform in much less expensive laboratory or semi-field assays needs to be ascertained. Once moth performance is better understood using various linked assays such as flight ability tests, wind tunnel flight performance tests, mating cage tests, field cage tests, and release–recapture tests, some of these assays might be routinely used in operational programs to appropriately and routinely assess (and ultimately improve) the quality of released moths [108].

The following research summarizes recent developments and the validation of simple bioassays that can be carried out in the laboratory and that had potential to be good surrogates for expensive and laborious field studies. A commercially available insect locomotion activity meter that contained both wild and sterile moths (in this study LBAM), and that were stimulated with repeatable pheromone puffs, indicated a significantly greater response of non-irradiated males than irradiated males [133]. A simple flight cylinder bioassay could detect differences in codling moth quality induced by various treatments whose effects could also be detected by more complex laboratory essays and field trials [134]. However, walk-in field cage bioassays proved to be a better predictor of male moth performance in an orchard than the flight cylinders [135]. Machine vision that records and analyzes insect behavior was used to track the flights of Australian painted apple moth males toward a calling female in a wind tunnel. The tracks of irradiated moths were different from those of untreated moths, although both arrived at the calling females [130]. Standard field cage tests routinely used to measure the sexual competitiveness of factory-reared tephritid fruit flies were validated and adapted for moths (LBAM as model species). The results indicated that sterile-male-only releases have the potential to increase the mating competitiveness of the released irradiated moths, but this conclusion requires additional experiments for confirmation [136].

In an AW-IPM program that has an IS component, F_1_ sterile males cannot be distinguished from wild fertile males, which makes monitoring the progress of such a program difficult. A cytological technique based on orcein and Giemsa stains was developed and could distinguish the adult F_1_ progeny of irradiated males from fertile males of six pest species from five lepidopteran families [137]. In a similar study with the Australian painted apple moth, the accuracy of the technique proved to be strongly correlated with male survival [138].

Trapping/monitoring in most Lepidoptera programs that have an SIT/IS component relies on the use of female pheromones to attract male moths. Trapping systems that would enable the sampling of female moths would offer the potential of monitoring the female moth populations in the field, their mating status, quality, and release distribution [124]. Female moth attractants are already used for the routine monitoring of codling moth populations [139], and efforts were made to develop female attractants for other Lepidoptera such as the diamondback moth (DBM) *Plutella xylostella* L. (Plutellidae). DBM females were significantly more attracted to conspecific larvae-infested cabbage and had significantly shorter flights in an arena with larvae-infested cabbage as compared with an arena with intact uninfested cabbage hosts, which indicates the potential of developing a brassica host-derived kairomone attractant [140].

Marking released sterile moths (e.g., by incorporating dyes into their diet or by external marking with fluorescent powders) is an important component of any program that has an SIT/IS component, as it enables the assessment of program progress [124]. However, problems of toxicity or reliability/misdiagnosis have instigated the development of other, more consistent approaches. These include a genetically engineered strain of pink bollworm with a heritable fluorescent marker [141] or the use of stable isotopes to distinguish wild from reared insects [142,143]. The stable isotopic labeling of seven lepidopteran species, namely (*C. cactorum*, *E. saccharina*, the oriental leaf worm *Spodoptera litura* (Fabricius) (Noctuidae), *E. postvittana*, *P. xylostella*, *L. botrana*, and *P. gossypiella* enabled the distinction of mass-reared from wild moths [144].

## 7. Challenges

As can be seen from this review, over the last 20 years, considerable progress has been made in the research on the applicability and improvement of SIT/IS for the control of lepidopteran pests, including the successful implementation of SIT programs against several major agricultural pests. However, there are still some challenges to further improve the applicability of SIT/IS.

Current programs using SIT/IS against lepidopteran pests rely on bisexual releases, but there are reasons to believe that male-only releases would bring significant economic benefits by decreasing operational costs and increasing the efficiency of the sterile males [145]. This has been well demonstrated in the SIT programs against the Mediterranean fruit fly (medfly), *Ceratitis capitata* (Wiedemann), using a genetic sexing strain that allowed the mass production and release of males only [146]. On the contrary, the results of field-cage experiments with several lepidopteran pests, such as the cactus moth, suggest that sterile females might have a positive impact on population suppression [12,147], thus casting doubts on the need for genetic sexing strains. However, experience with the Mediterranean fruit fly shows that a clear conclusion cannot be reached without large-scale field tests that would compare the efficiency of male-only versus bisexual releases for the control of lepidopteran pests.

In Lepidoptera, a pure genetic sexing system has been developed in only two species: the silkworm *B. mori* [148,149] and the flour moth *E. kuehniella* [150]. This sexing system is based on the construction of a balanced lethal strain generating transheterozygous males for two sex-linked recessive lethal mutations. When the males are mated to wild-type females, the F_1_ generation consists almost exclusively of male progeny. However, this system is impractical for SIT/IS programs. It requires (i) the mass rearing of two colonies, (ii) the manual separation of sexes in both colonies before crosses producing male-only progeny, and (iii) the regular checking of the genetic structure of the balanced lethal strain to prevent its breakdown [56].

**Figure 9:**
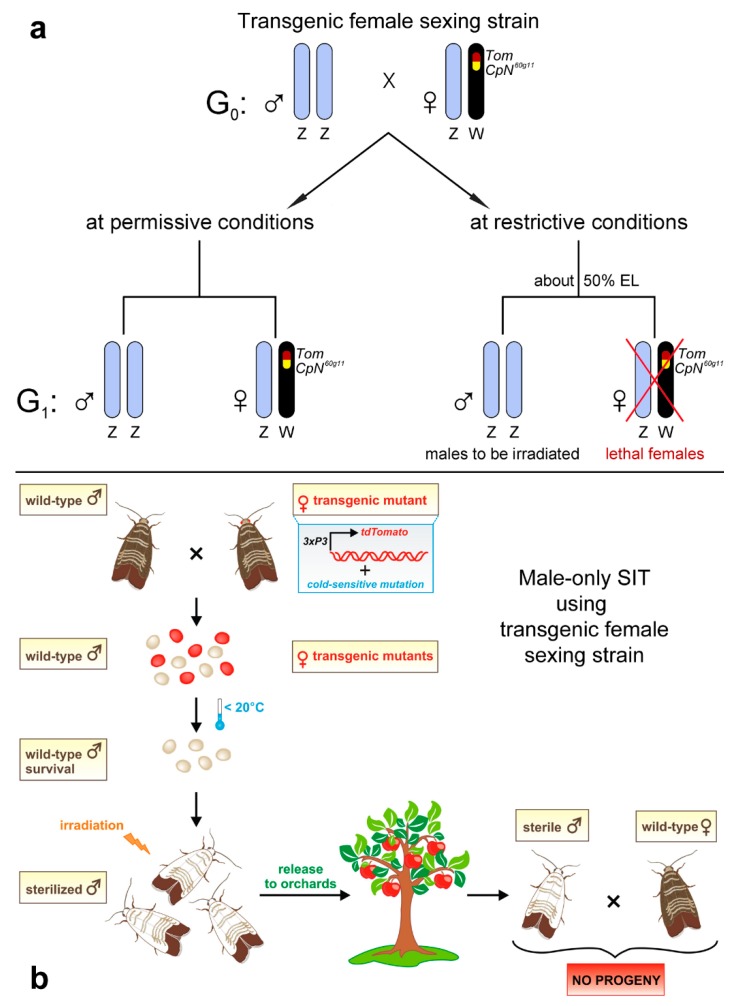
Transgenic female sexing strain proposed for the control of codling moth populations using the sterile insect technique (SIT) [145,153]. (**a**) Scheme of genetic sexing strain based on the use of transgenic females that possess an insert in their W chromosome, containing the *tdTomato* marker gene (red) and the codling moth ortholog (*CpN^60g11^*; yellow; R. Čapková Frydrychová and F. Marec, unpublished) of the dominant cold-sensitive mutant allele of the *Notch* gene of *Drosophila melanogaster*, *N^60g11^*. EL, embryonic lethality. Z and W, sex chromosomes. (**b**) Scheme of male-only SIT using transgenic female sexing strain. Wild-type males are mated with transgenic females, expressing the red fluorescent protein in their eyes under the *3xP3* promoter. The obtained egg collections are exposed to a temperature below 20 °C, resulting in the death of all transgenic female embryos. Non-transgenic male-only offspring are kept until adulthood, sterilized by ionizing radiation, and released to apple orchards.

New opportunities for genetic sexing emerged along with advances in genetic technologies, especially in transgenesis and gene editing. In Lepidoptera, the first germline transformations were performed in the pink bollworm and in the silkworm using a *piggyBac* transposable element [151,152]. Based on this success, it has been proposed to construct genetic sexing strains using a transgenic approach so that the females carry a transgene with a dominant conditional lethal mutation in their W chromosome that would be expressed in embryogenesis. Such strains, if their eggs are kept at restrictive conditions, would produce non-transgenic male-only progeny that could be irradiated and released (Figure 9a,b) [145]. The development of such strains was initiated in the codling moth [153], but this work was later discontinued due to the low efficiency of transgenesis in this species. That this approach is feasible has recently been demonstrated in the silkworm by the successful insertion of a transgene carrying the EGFP (enhanced green fluorescent protein) reporter into the W chromosome [154]. Furthermore, new transgenic strains providing a conditional sex-specific lethality were constructed in the silkworm [155] and in two pest species: the diamondback moth and the pink bollworm [156]. In these strains, conditional female lethality is controlled by the tetracycline-repressible transactivator (tTAV) protein, which is expressed only in females due to the female-specific spliced region of the pink bollworm *doublesex* gene, *Pgdsx*. Methods of gene editing appear to be even more promising for the development of genetic sexing strains than transgenesis, because they allow targeted mutagenesis. Although these methods have not yet been used for genetic sexing in Lepidoptera, particularly the CRISPR/Cas9 (clustered regularly interspaced short palindromic repeats/CRISPR-associated protein 9) technique has already been successfully established in several species and used to study gene functions [157,158].

A detailed knowledge of the sex-determining pathway could greatly facilitate the development of genetic sexing strains that are suitable for the mass rearing of lepidopteran pests. However, little is known about the mechanism of sex determination in Lepidoptera except for the silkworm *B. mori* [16]. In this model species, a homologue of the *Drosophila doublesex* (*dsx*) gene, *Bmdsx*, was found to be sex-specifically spliced and confirmed to control sexual differentiation, as does the *Drosophila dsx* gene [159,160]. This bottom gene of the sex-determining cascade in *Drosophila* seems to be the only conserved gene of this cascade in Lepidoptera [161,162,163]. In the silkworm, sex determination depends on the presence or absence of the W chromosome, which carries a dominant female-determining factor (*Fem*) that promotes femaleness, irrespective of the number of Z chromosomes present in the genome [164]. Recently, a surprising discovery has been made that *Fem* is not a protein-coding gene, as originally assumed, but rather a W-encoded small RNA named *Fem* piRNA. The *Fem* piRNA downregulates the expression of a Z-linked gene, *Masculinizer* (*Masc*), which promotes male development through male-specific splicing of the *Bmdsx* gene in the absence of the W chromosome [165].

However, it is not yet known whether the *Fem* piRNA-*Masc* sex-determining pathway is conserved in other lepidopteran species with the W chromosome. For example, recent results of cross-hybridization between geographical subspecies of wild silkmoths *S. cynthia* clearly suggest that the W chromosome plays no role in sex determination [166]. It also remains to be clarified whether *Masc* plays a role in species with a Z0/ZZ sex chromosome system that are thought to have a Z-counting mechanism of sex determination [16,18]. Thus, the *dsx* gene, whose function appears to be conserved in many insects, including Lepidoptera [160,167], is currently the only available target for the development of genetic sexing strains in lepidopteran pests.

The use of the SIT/IS has played and will continue to play a critical role in AW-IPM campaigns against key lepidopteran pests, and this is becoming even more pertinent in view of their dramatic geographic range expansion in recent decades [168]. Despite the progress made in recent years with the identification of factors that affect the field performance of sterile male moths and the development of methods and tools to assess field performance, there are several gaps that remain to be addressed, especially with respect to the rearing and release processes that may affect the quality and field performance of the moths. There are several lepidopteran species that currently cannot be reared, and for which an artificial diet is not available, such as the lesser date moth *Batrachedra amydraula* Meyrick, the litchi stem-end borer *Conopomorpha sinesis* Bradley, and the cocoa pod borer *Conopomorpha cramerella* (Snellen). In addition, there is a need for the improved maintenance of colonies based on the selection and preservation of desirable traits, better collection, irradiation, handling, transport, and release methods, and a better understanding of the efficacy of male-only releases compared with bi-sex releases.

## 8. Conclusions

The successful implementation of SIT/IS in control programs against several key pests of the phylogenetically distant clades of Lepidoptera (see above) has clearly demonstrated the high efficiency and wide applicability of these environment-friendly strategies for the control of lepidopteran pests. Significant advances have also been achieved in the knowledge of Lepidoptera genetics and genomics and in understanding the nature of the high resistance of Lepidoptera to ionizing radiation and the principle of inherited sterility. In addition, extensive research has provided valuable knowledge, tools, and methods that are needed for the mass rearing and quality control of lepidopteran pests, which promises to improve the performance of released insects in the field. All these achievements paved the way for further expanding the SIT/IS to control other candidate pest species of the order Lepidoptera.
